# Protocol for generation of transmitochondrial cybrids under pyruvate/uridine-supplemented conditions using a microfluidic device

**DOI:** 10.1016/j.xpro.2025.103953

**Published:** 2025-07-17

**Authors:** Ken-Ichi Wada, Kazuo Hosokawa, Yoshihiro Ito, Mizuo Maeda, Yui Harada, Yoshikazu Yonemitsu

**Affiliations:** 1 Institute for Materials Chemistry and Engineering, Kyushu University, 744 Motooka, Nishi, Fukuoka 819-0395, Japan; 2 Graduate School of Pharmaceutical Sciences, Kyushu University, 3-1-1 Maidashi, Higashi, Fukuoka 812-8582, Japan; 3 RIKEN, 2-1 Hirosawa, Wako, Saitama 351-0198, Japan

**Keywords:** Cell culture, Single cell, Genetics, Biotechnology and bioengineering

## Abstract

Under conventional approaches, selective culture with a pyruvate/uridine (PU)-free medium is essential for generating transmitochondrial cybrids. Here, we present a protocol for generating transmitochondrial cybrids using a microfluidic device, which works even under PU-supplemented conditions. We describe steps for preparation of the microfluidic device, partial cell fusion (mitochondrial transfer), and harvest of transmitochondrial cybrids. We then detail procedures for confirmation of mtDNA repopulation in cybrids and mtDNA typing by restriction fragment length polymorphism (RFLP) analysis.

For complete details on the use and execution of this protocol, please refer to Wada et al.^1^

## Highlights

• Protocol for the generation of transmitochondrial cybrids using a microfluidic device
• Instructions for the fabrication of a microfluidic device
• Steps for mitochondrial transfer between single cells using a microfluidic device
• Guidance on harvesting cybrids under pyruvate/uridine-supplemented conditions

## Abstract (editor highlights)

Publisher’s note: Undertaking any experimental protocol requires adherence to local institutional guidelines for laboratory safety and ethics.

## Graphical abstract

**Figure:**
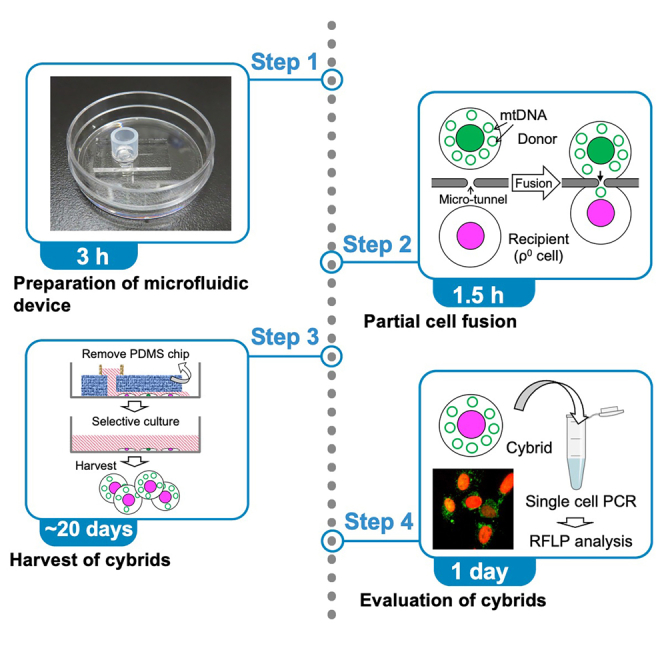

## Before you begin

This protocol describes the specific steps for generating transmitochondrial cybrids using a microfluidic device. Before you begin, it is necessary to prepare the puromycin-resistant cells (donor) and mtDNA-less (ρ^0^) cells (recipient).^2^ Since the microfluidic device is fabricated by soft lithography,^3^ you need to prepare the master mold for PDMS microfabrication.

### Preparation of Pur^R^ donor and ρ^0^ recipient cells

**Timing: 2–3 months**

This section describes the preparation of donor and recipient cells. Use of a TK-deficient cell line is essential for generation of ρ^0^ recipient cells. In this protocol, donor and recipient ρ^0^ cells are generated from HeLa and 143B cells, respectively.

1. Prepare ρ^0^ recipient cells.
  a. Culture 143B cells with EtBr medium until their mtDNA completely disappear (usually, 30-60 days).
    ***Note:*** During this culture, replace the EtBr medium every day and keep the cells with < 60% confluency with proper passages. In every passage, you can check the existence of mtDNA using the redundant cells by PCR method described in the Step-by-step method details.
  b. Further culture the cells with PU medium for >1 month.
  c. Confirm no mtDNA recovery by PCR (see step-by-step method details, Evaluation 2).
    **CRITICAL:** If mtDNA is recovered, do not use the cells thereafter, and redo from step 1a using a new cell/stock. See also troubleshooting 1.
  d. If necessary, introduce certain marker genes into the established ρ^0^143B cells.
2. Prepare mitochondrial donor and recipient cells.
  a. Stably transfect puromycin resistant gene (Pur^R^) or Hygromycin resistant gene (Hyg^R^) into HeLa or ρ^0^143B cells with a standard protocol.
  b. If necessary, further introduce certain marker genes.

**CRITICAL:** The donor and recipient cells must be TK(+) and TK(−), respectively. Also, they must be introduced different kind of resistant gene. In this protocol, HeLa and ρ^0^143B cells are stably transfected with H2B-EGFP-IRES-Pur and H2B-mCherry-IRES-Hyg, respectively.

**Pause Point:** Generated ρ^0^ cells (at least ρ^0^143B cells) can be cryopreserved with a standard method in −80 °C. For long-term preservation, store in gas phase of liquid nitrogen.

### Preparation of master mold

**Timing: 1 week**

**Figure 1:**
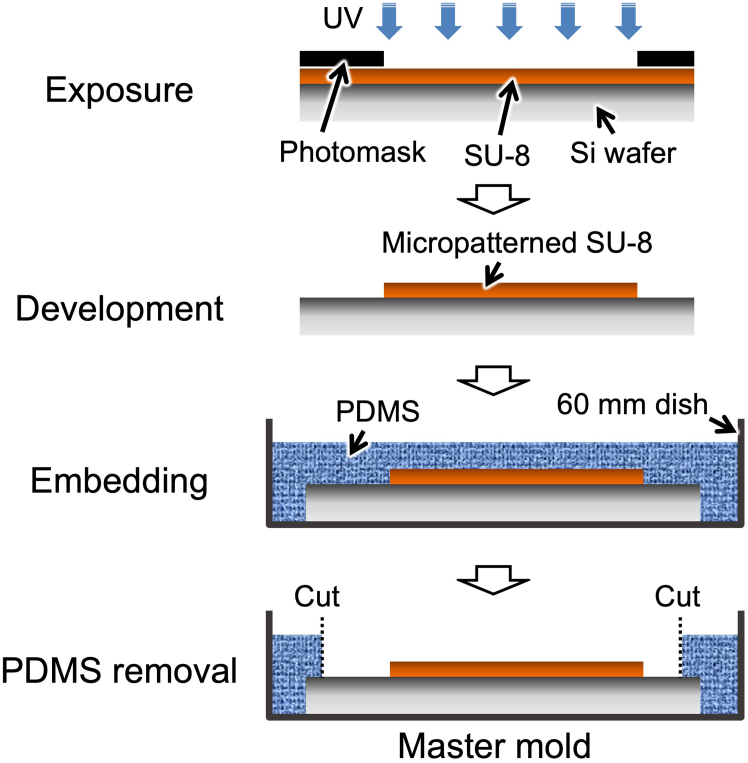
Schematic illustration of procedures for master mold fabrication

This section describes how to fabricate the master mold (Figure 1). This process should be carried out in a clean room (yellow room).

**Figure 2:**
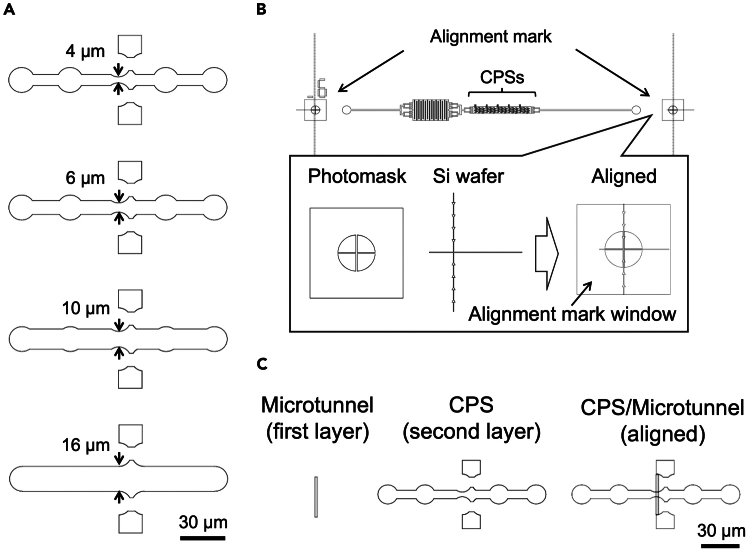
Summary of photomask designs (A) Variation of cell pairing structure (CPS). The microtunnel length is defined by the distance between cell pockets indicated by arrows. Bar: 30 μm. (B) Summary of the alignment between Si wafer, first layer (microtunnels) and second layer (CPSs). The alignment mark on Si wafer can be seen through the alignment mark window. The arrowheads pattern helps to identify the Y axial position for alignment. (C) Magnified view of CPS/microtunnel design. The CPS with a short (4 μm-length) microtunnel is shown. For details of photomask designs, see Figures 1, 2, and 3 and the deposited CAD data (dwg files: Mask #4, #20 and #27). Bar: 30 μm.
  b. Request fabrication of glass photomask (chrome mask) to a manufacturer (e.g., System advance Co., Ltd.). Usually, it takes several days for the delivery of the photomask.

3. Prepare photomask.
  a. Draw the designs for alignment mark on Si wafer, first layer of microtunnels, and second layer of cell paring structures (CPSs)/main channel using a computer-aided design (CAD) program (Figure 2). Alternatively, you can utilize the deposited CAD data (dwg files: Mask #4, #20 and #27, Figures S1, S2, and S3).

**CRITICAL:** Due to the smallness of the microtunnel, use of glass photomask is strongly recommended. Film photomask would not be applicable.

4. Cut Si wafer into 32 × 32 mm square pieces.

***Note:*** The deposited CAD data are adjusted to this Si wafer size; however, you can change the size according to your demand.

5. Clean Si wafer.
  a. Sonicate with IPA for 1 min.
  b. Sonicate with ultrapure water for 1 min.
6. Dry Si wafer.
  a. Spin dry at 3,000 rpm for 1 min.
  b. Bake at 140°C for 1 h.

***Note:*** For Si wafer baking, use a clean dry oven. Do not share for PDMS baking (curing). Adsorption of PDMS reagent vapor to Si wafer causes poor adhesion of SU-8.

7. Apply plasma etching with O_2_ (20 Pa), 150 W for 15 min.
8. Spin coat the Si wafer with S1805 at 3,000 rpm for 25 s.
9. Bake at 120°C for 1 min.
10. Set the photomask (alignment mark) and the Si wafer to mask aligner.
11. Expose UV light for 5 s.
12. Develop the Si wafer.
  a. Gently shake in NMD-3 followed by ultrapure water for 1 min each.
  b. Spin dry at 3,000 rpm for 1 min.
  c. Bake at 120°C for 3 min.
13. Engroove alignment mark.
  a. Apply plasma etching with O_2_ (6 Pa), 100 W for 1 min.
  b. Apply plasma etching with SF_3_ (20 Pa), 200 W for 1 min.
  c. Apply plasma etching with O_2_ (20 Pa), 150 W for 15 min.
14. Spin coat the Si wafer with SU-8 5 at 7,000 rmp for 15 sec to make a 2 μm-thick layer (first layer).
15. Bake at 90°C for 1 h.
16. Set the Si wafer and photomask (microtunnels) to mask aligner with fine alignment.
17. Expose UV light for 10 s (first exposure).
18. Develop the Si wafer.
  a. Gently shake in SU-8 developer for 5 min.
  b. Gently shake in IPA for 2 min.
  c. Spin dry at 3,000 rpm for 1 min.
19. Spin coat the Si wafer with SU-8 25 at 2,500 rpm for 15 sec to make a 26–28 μm-thick layer (second layer).
20. Bake at 90 °C for 1 h.
21. Set the Si wafer and photomask (CPSs/main channel) to mask aligner with fine alignment.
22. Expose UV light for 10 s (second exposure).
23. Develop the Si wafer in the same way as in step 18.
24. Bake the Si wafer at 140°C for 10 min.
25. Cool slowly to approximately 20°C–25 °C.
  a. Stop heating.
  b. Leave for several hours keeping the oven door closed.
26. Check the fabricated micropattern (CPSs). troubleshooting 2.
27. Fluorinate the Si wafer (optional).
  a. Apply plasma process with CHF_3_/O_2_ (20 Pa), 200 W for 5 min.
  b. Apply plasma process with CHF_3_ (20 Pa), 200 W for 2 min.

***Note:*** Fluorinate process provides low adhering of PDMS to the master mold.

28. Embed the Si wafer in PDMS.
  a. Mix well PDMS oligomer and crosslinking prepolymer in a ratio of 10:1 (w/w).
  b. Place the master mold in a 60 mm dish and pour approximately 6 g of PDMS mixture.
  c. Vacuum until bubbles disappear.
  d. Cure PDMS at 65°C for >1 h.
  e. Cut PDMS along inside edge of the Si wafer and peel away to open SU-8-micropatterned region.

**CRITICAL:** Conditions of spin coat, UV light exposure time, and alignment accuracy are critical for fabrication of fine master mold. These must be optimized carefully.

## Key resources table

**Table:** 

REAGENT or RESOURCE	SOURCE	IDENTIFIER
**Chemicals, peptides, and recombinant proteins**		

SYLGARD 184 Silicone Elastomer kit (PDMS oligomer/crosslinking prepolymer)	The Dow Chemical Company	N/A
SU-8 5/25	Kayaku Advanced Materials, Inc.	N/A
SU-8 developer	MicroChem	N/A
MICROPOSIT S1805 positive photoresist	The Dow Chemical Company	S1805
NMD-3	TOKYO OHKA KOGYO Co., Ltd.	N/A
Isopropyl alcohol (IPA)	FUJIFILM Wako Pure Chemical Corporation	166-04836; CAS: 67-63-0
DMEM (high glucose) with L-glutamine and phenol red (DMEM)	FUJIFILM Wako Pure Chemical Corporation	044–29765
Fetal bovine serum (FBS)	Life Technologies	10437–028
Sodium pyruvate (100 mM) (pyruvate)	Thermo Fisher Scientific	11360070; CAS: 113-24-6
Uridine	Sigma-Aldrich	U6381; CAS: 58-96-8
5′-Bromo-2′-deoxyuridine (BrdU)	Sigma-Aldrich	B5002
GenomeONE-CF (HVJ-E)	ISHIHARA SANGYO KAISHA, Ltd.	Discontinued production
Y-27632	Sigma-Aldrich	688000; CAS: 146986-50-7
Quant-iT PicoGreen dsDNA reagent (PicoGreen)	Thermo Fisher Scientific	P11495; CAS: 177571-06-1
EtBr solution, 10 mg/mL (EtBr)	Nippon Gene Co., Ltd.	315–90051
KOD -Plus- Ver.2 (KOD, 10×PCR buffer, dNTPs, MgSO_4_)	TOYOBO Co., Ltd.	KOD-211
Proteinase K solution	Kanto Chemical Co., Inc.	34060–96
*Ssp*I-HF (*Ssp*I)	New England Biolabs	R3132S
1 M Tris-HCl (pH 8.0)	Nippon Gene Co., Ltd.	312–90061
Tween 20	Nacalai Tesque, Inc.	35624–02
Tris-Borate-EDTA buffer (10x)	Nacalai Tesque, Inc.	35440–31
Agarose	Nacalai Tesque, Inc.	01163–76
PBS(−)	Nacalai Tesque, Inc.	14249–95

**Deposited data**		

CAD data/alignment mark (Mask #4)	This paper	Mendeley Data: https://doi.org/10.17632/zh7y93h78t.1
CAD data/first layer, second layer (Mask #20)	This paper	Mendeley Data: https://doi.org/10.17632/zh7y93h78t.1
CAD data/first layer, second layer (Mask #27)	This paper	Mendeley Data: https://doi.org/10.17632/zh7y93h78t.1

**Experimental models: Cell lines**		

HeLa	RIKEN BRC	RCB0007
143B/TK^-^neo^R^ (143B)	RIKEN BRC	RCB0701

**Oligonucleotides**		

Primer: human mtDNA forward: ATCGCTCACACCTCATATCC	Wada et al.^1^	N/A
Primer: human mtDNA reverse: ATCGCTCACACCTCATATCC	Wada et al.^1^	N/A
Primer: EGFP forward: CCGACCACATGAAGCAGCAC	Wada et al.^1^	N/A
Primer: EGFP reverse: TCACGAACTCCAGCAGGACC	Wada et al.^1^	N/A
Primer: mCherry forward: TCACGAACTCCAGCAGGACC	Wada et al.^1^	N/A
Primer: mCherry reverse: TCTTGGCCTTGTAGGTGGTC	Wada et al.^1^	N/A

**Recombinant DNA**		

pCAG-H2B-EGFP-IRES-Pur	Wada et al.^4^	N/A
pCAG-H2B-mCherry-IRES-Hyg	Wada et al.^4^	N/A

**Software and algorithms**		

AutoCAD	Autodesk, Inc.	N/A
LuminaVision	Olympus Corporation	Ver. 3.37.0

**Other**		

Clean room/booth	N/A	Yellow light condition
Photo mask aligner	Union Optical Co., Ltd.	PEM-800
Reactive ion etching (RIE) – plasma etching system	Samco, Inc.	Performing plasma process of O_2_, SF_3_, and CHF_3_ gas.
Spin coater	Mikasa Co., Ltd.	MS-B100
Dry oven (for Si wafer)	N/A	N/A
Dry oven (for PDMS)	N/A	N/A
Polished Si wafer (Si wafer)	N/A	N/A
Chrome glass mask (photomask)	System Advance Co., Ltd.	N/A
Silicone tube, 5 mm inner diameter	N/A	N/A
60 mm dish	Falcon	353002
35 mm dish	Falcon	353001
Tabletop clean bench	AS ONE Corporation	CT-600UVAX-L
Electric scale	N/A	Put in tabletop clean bench
Biopsy trepan, 2.0 mm diameter	KAI MEDICAL	BPP-20F
Centrifuge	Kubota Corporation	Model 3740 equipped with swing rotor (SF-242) or plate rotor (PF-206).
Reverse fluorescent microscope	Olympus Corporation	IX81 equipped with CCD camera (QImaging, ROLERA-XR).
Reverse fluorescent microscope	KEYENCE	BZ-X800
Time-lapse microscope	Nikon	BioStudio-mini, equipped with ×10 objective lens.

## Materials and equipment

**Table:** PU medium

Reagent	Final concentration	Amount
DMEM	N/A	40 mL
FBS	10%	4 mL
100 mM pyruvate	1 mM	400 μL
5% uridine	1 μg/mL	40 μL

Store at 4°C up to 2 weeks.

• FBS: immobilized with heating at 56°C for 30 min.

Store at −20°C.

• Uridine: dissolve with milliQ water to make 5% (w/v) then sterilize with 0.22 μm filter.

Store at −20°C.

**Table:** Fusion medium

Reagent	Final concentration	Amount
DMEM	N/A	400 μL
HVJ-E suspension	N/A	10 μL
5 mM Y-27632	100 μM	8 μL

Prepare just before use.

• HVJ-E suspension: add 260 μL ice cooled DMEM to 1 vial of HVJ-E and pipette to make HVJ-E suspension. It is recommended to divide the HVJ-E suspension into 10 μL stocks in sterilized 1.5 mL tube.

Store at −70°C.

• Y-27632: dissolve 1 mg Y-27632 with 590 μL sterilized milliQ water to make 5 mM stock.

Store at −20°C.

**Table:** Proteinase K buffer

Reagent	Final concentration	Amount
MilliQ water	N/A	36 mL
1 M Tris-HCl (pH 8.0)	50 mM	2 mL
10% Tween 20	0.5%	2 mL
Total	N/A	40 mL

Store at approximately 20°C–25°C up to several months.

• Tween 20: dissolve with milliQ water to make 10% (v/v) solution.

Store at approximately 20°C–25°C up to several months.

**Table:** *Ssp*I working solution

Reagent	Final concentration	Amount
MilliQ water	N/A	10 μL
10× Cut Smart	1.25×	70 μL
*Ssp*I (20,000 U/mL)	0.6 U/μL	2.5 μL
Total	N/A	82.5 μL

Prepare just before use.

### BrdU solution

• Add 20 mL PBS to 100 mg BrdU to make 5 mg/mL solution, then sterilize with 0.22 μm filter.

Store at −20°C.

### Puromycin solution

• Dissolve with sterilized milliQ water to make 10 mg/mL solution.

Stored at −20°C.

### Culture medium

• Add 4 mL FBS to 40 mL DMEM.

Stored at 4 °C up to 2 weeks.

### ×0.1 PU medium

• Ten times dilute PU medium with culture medium.

Stored at 4°C up to 2 weeks.

### EtBr medium

• Add EtBr solution (final concentration: 50 ng/mL) to PU medium.

Store at 4°C up to 2 weeks. EtBr solution must be handled with extreme caution not to contact to skin.

### Pur selection medium

• Add 8 μL puromycin solution (final concentration: 2 μg/mL) to 40 mL PU medium. Prior to experiment, puromycin concentration should be optimized.

Store at 4°C up to 2 weeks. For optimization, determine minimal pur concentration that all target cells die within 3–5 days.

### BrdU selection medium

• Add BrdU solution (final concentration: 50 μg/mL) to culture medium.

Store at 4°C up to 2 weeks.

### PU/BrdU selection medium

• Add BrdU solution (final concentration: 50 μg/mL) to PU medium.

Store at 4°C up to 2 weeks.

### PicoGreen medium

• Dilute PicoGreen reagent 1:500 with growth medium.

Prepare just before use.

### Proteinase K working solution

• Add 5 μL proteinase K solution (final concentration: 200 μg/mL) to 500 μL proteinase K buffer.

Prepare just before use.

## Step-by-step method details

### Preparation of the microfluidic device

**Timing: 3 h**

**Figure 3:**
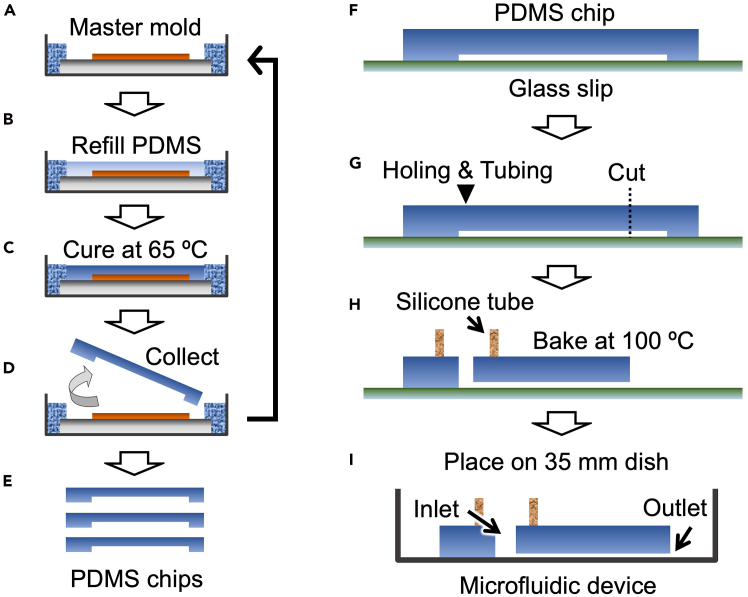
Schematic illustration of procedures for microfluidic device preparation (A) Empty master mold. (B–D) Procedure for PDMS chip preparation (steps 2–7). (E) Collected PDMS chips. (F-I) Procedure for the microfluidic device preparation (steps 10–14).

**Figure 4:**
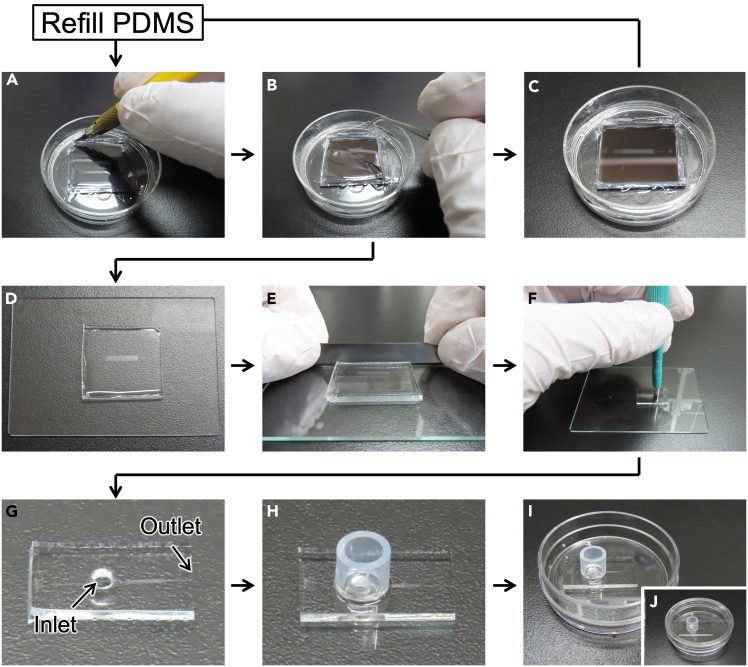
Images of microfluidic device preparation (A) Cut cured PDMS (step 6). (B) Peel off PDMS (step 7). (C) Empty master mold. It is recommended to store the master mold in empty to avoid sticking of PDMS to Si wafer. (D) Place the PDMS chip on a clean glass slip (step 8). (E–G) Trim and punch out to make outlet and inlet (step 10). (H) Bond silicone tube to make an inlet reservoir (steps 11–13). (I) Place the PDMS chip on a 35 mm dish (step 14). (J) Microfluidic device can equip the lid because of no connection to any external machines. PDMS chips shown in G-I are not identical.

This section describes how to prepare the microfluidic device (Figures 3 and 4). This process can be carried out in a conventional biological laboratory, but use of desktop clean bench is recommended.

1. Prepare inlet reservoir material.
  a. Cut a 5 mm-inner diameter silicone tube to 5–7 mm.
  b. Stored the cut tubes in 100% ethanol.
  c. Air-dry the tubes before use.

***Note:*** Storing cut silicone tube in ethanol is for wash and sterilization.

2. Mix well PDMS oligomer and crosslinking prepolymer in a ratio of 10:1 (w/w).

***Note:*** Mixed PDMS can be maintained uncured for several hours at approximately 20°C–25°C.

3. Pour PDMS mixture to master mold to approximately 2 mm-thick (1.4–1.6 g).
4. Vacuum until bubbles disappear.
5. Cure PDMS at 65°C for >1 h.
6. Cut PDMS along inside edge of the Si wafer.
7. Peel off micropattern-imprinted PDMS (PDMS chip) from master mold.
8. Place the PDMS chip on a clean glass slip.
9. Repeat steps 3–7 to prepare a number of PDMS chips if necessary.

***Note:*** At least several hundred PDMS chips can be reproduced from one master mold. The PDMS chip can be stored putting on a glass slip, but storage period should be up to a few months because of a risk for sticking to glass slip.

10. Punch out 2 mm-diameter hole using a biopsy trepan and cut PDMS chip to make inlet and outlet, respectively.
11. Apply uncured PDMS mixture to one side cut surface of silicone tube prepared in step 1. troubleshooting 3.
12. Put the silicone tube at the region of inlet hole to make inlet reservoir.
13. Bake at 100 °C for >1 h to bond the silicone tube and completely cure the PDMS chip.

***Note:*** This process is partly for sterilization. Bake the PDMS chip for 12–24 h if necessary.

14. Place the resulting PDMS chip on 35 mm culture dish to prepare the microfluidic device.
15. Put the lid on.

**CRITICAL:** Do not redo PDMS chip placing, because cell adhesion is interfered at the PDMS-contact region. Additionally, do not apply plasma process to PDMS chip for inducing tight adhesion with culture dish. Reversible bonding is required in this protocol.

**Pause Point:** Prepared microfluidic devices can be stored under a normal atmosphere for at least several weeks.

### Partial cell fusion

**Timing: 1.5 h**

**Figure 5:**
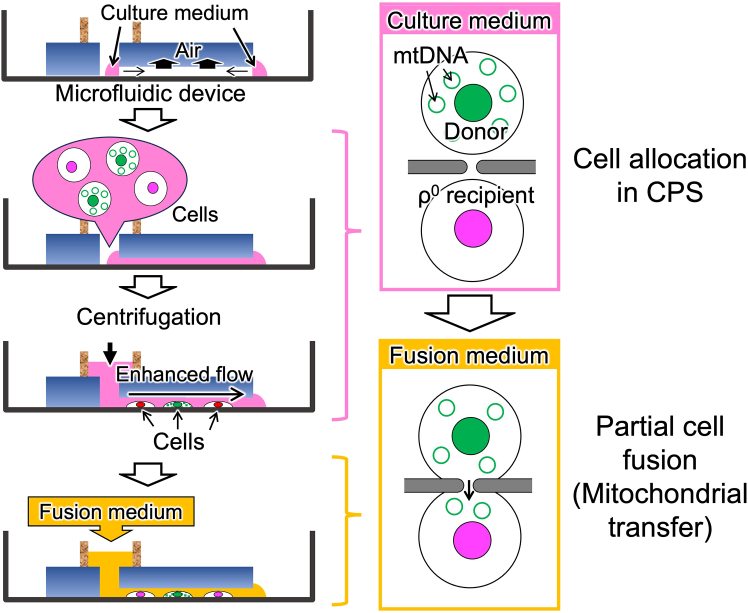
Schematic illustration of procedures for cell fusion through a microtunnel In this scheme, mitochondria are omitted.

This section describes how to achieve partial cell fusion (fusing cells through a microtunnel) and transfer mitochondria to ρ^0^ cells (Figure 5). Procedures should be carried out in a clean bench when lid of the microfluidic device is opened.

16. Prepare donor and ρ^0^ recipient cells with approximately 60% confluency in a 35 mm dish.
17. Fill microchannel with culture medium.
  a. Place microfluidic device in a vacuum chamber to degas at approximately −0.06 MPa for > 10 min.
  b. Immediately after taking microfluidic device out, fill inlet and outlet with a drop of culture medium.

***Note:*** The microchannel is filled with the applied culture medium within several minutes because of air adsorption by surrounding PDMS substrate.^5^

18. Harvest the cells from culture dish by trypsinization and mix them to make a cell suspension of 0.5–1 × 10^6^ cells/100 μL culture medium.
19. Inject 10 μL cell suspension into the inlet hole.
20. Gently add 100 μL culture medium to the inlet reservoir.
21. Put the lid on and set in a swing bucket rotor fixing with a double-sided tape.
22. Centrifuge at 440 × *g* for 1 min and confirm cell allocation in CPSs.

***Note:*** Centrifugation transiently increases flow velocity in the microchannel, leading to cell allocation in CPSs. If the cell allocation is insufficient (usually, 70%–90% CPSs are occupied with 1:1 cells), redo centrifugation after pipetting the applied cell suspension. If cell aggregations are formed in the microchannel, redo from step 17 using a new microfluidic device. See also troubleshooting 4.

23. Aspirate remaining culture medium from the inlet reservoir leaving a drop on the inlet hole.

**Figure 6:**
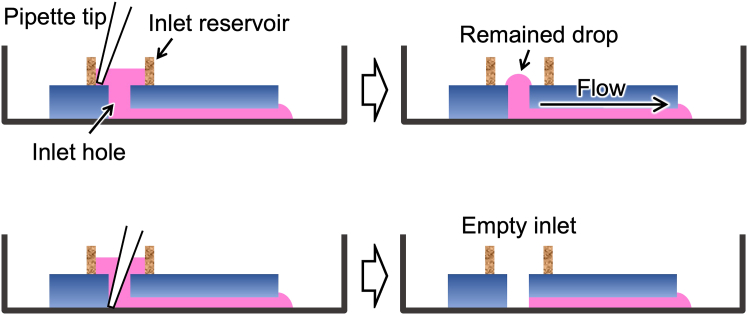
Schematic illustration of ways to aspirate inlet medium Upper: When medium is aspirated inserting pipette tip along with the inlet reservoir inner wall, a convex drop remains on the inlet hole. In this situation, microfluidic flow is maintained due to its surface tension. Lower: When medium is aspirated inserting pipette tip into inlet hole, all medium is removed from the inlet. In this situation, although microfluidic flow transiently stops, you can complete inlet medium exchange with a single pipetting. After applying new medium, microfluidic flow recovers.

**CRITICAL:** Do not aspirate all culture medium. A convex drop at the inlet hole maintains gentle flow due to its surface tension, keeping cells trapped in the CPSs. See Figure 6.

24. Inject 50–100 μL fusion medium into the inlet hole.

***Note:*** To replace remaining culture medium with fusion medium, insert tip of micropipette to the bottom of inlet hole.

25. Place the microfluidic device in a CO_2_ incubator for 1 h.
26. Confirm cell fusion (optional).

***Note:*** Cell fusion through a microtunnel can be detected by observation of cytoplasmic components in the cells. For details, please refer to our previous publications.^1^^,^^4^^,^^6^^,^^7^^,^^8^ See also troubleshooting 5.

### Harvest of transmitochondrial cybrids

**Timing: 2–3 weeks**

**Figure 7:**
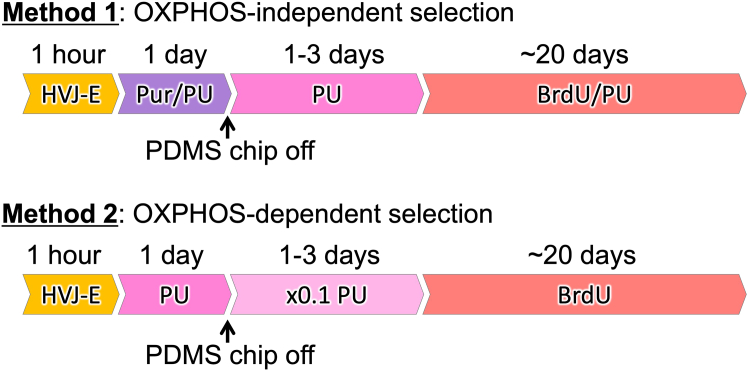
Summary of two methods for cybrid selection

**Figure 8:**
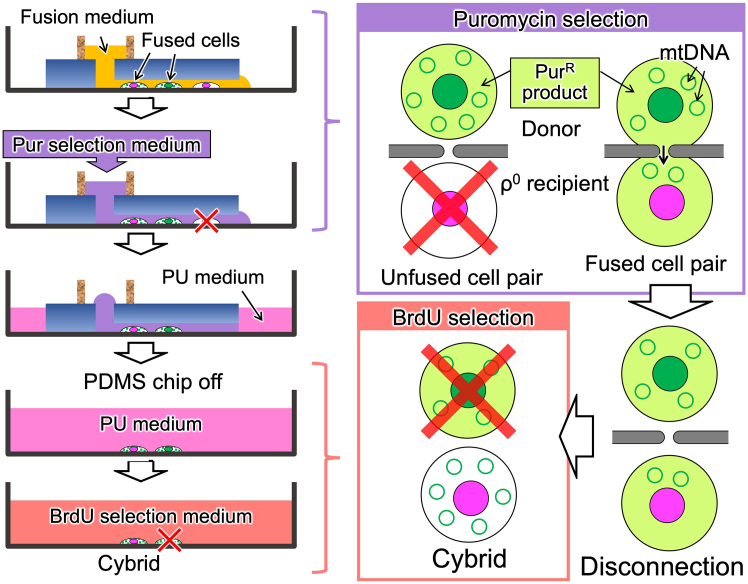
Schematic illustration of procedure for Method 1 In this scheme, mitochondria are omitted.

This section describes two methods for selective harvest of the resulting cybrids from the microfluidic device (Figure 7). Method 1 is a two-step selection, in which the unfused ρ^0^ cells are selectively removed by puromycin selection medium in the microfluidic device (first selection), subsequently, the cells containing donor nuclei (i.e., TK gene-present cells) are eliminated by PU/BrdU selection medium (second selection) (Figure 8). Method 2 is a one-step selection using the BrdU selection medium. This method is basically equivalent to a conventional transmitochondrial cybrid selection.

27. Aspirate all medium from the inlet reservoir/hole.
28. Add 100 μL Pur selection medium (Method 1) or PU medium (Method 2) to the inlet reservoir and put the lid on.
29. Culture in a CO_2_ incubator for 1 day.

***Note:*** During this culture, the fused cells are disconnected to recover into single cells. For details, please refer to the Expected outcomes in this protocol and our previous publication.^1^^,^^6^^,^^7^

**CRITICAL:** Prior to the experiment, conditions (puromycin concentration and exposure time) must be carefully optimized. To explore the conditions, perform the first selection using the microfluidic device being introduced with either only ρ^0^ recipient or donor cells, and confirm their extinction or survival, respectively. As long as satisfying this condition, use of minimal pur concentration is recommended.

30. Remove PDMS chip.
  a. Aspirate remaining puromycin selection medium leaving a little in the inlet hole.
  b. Add 1–1.5 mL PU medium outside the PDMS chip.
  c. Carefully remove the PDMS chip and gently shake to spread the PU medium.
  d. Replace with 1.5–2 mL PU medium (Method 1) or ×0.1 PU medium (Method 2).
  e. Put the lid on and place in a CO_2_ incubator.
  f. Culture for 1–3 days.

**CRITICAL:** Do not aspirate all medium from the inlet in removing PDMS chip. In that case, the cells frequently die due to quick evaporation of culture medium after removing the PDMS chip.

31. Continue cell culture with PU/BrdU selection medium (Method 1) or BrdU selection medium (Method 2) until colony appears.
32. Harvest the colony and expand with proper passages using the PU/BrdU selection medium (Method 1) or BrdU selection medium (Method 2) to obtain enough amount of transmitochondrial cybrids.

### Evaluation 1 (confirmation of mtDNA repopulation in cybrids)

**Timing: 30 min**

This section describes a simple method for confirming mtDNA repopulation in the resulting cybrids based on fluorescent observation.

33. Prepare the resulting transmitochondrial cybrids in a 35 mm dish.
34. Replace with 1-2 mL of PicoGreen medium and incubate for 15 min in a CO_2_ incubator.
35. Observe the cybrids using an inverted fluorescence microscope without fixation.

### Evaluation 2 (mtDNA typing by RFLP analysis)

**Timing: 3 h**

This section describes PCR-based restriction fragment length polymorphism (RFLP) analysis for mtDNA typing of HeLa and 143B cells. The PCR products can be prepared from both bulk and single cell samples.

36. Prepare PCR template (bulk sample).
  a. Extract 2-5 × 10^5^ cells with 500–1000 μL proteinase K working solution at 55°C for > 6 h.
  b. Heat at 95 °C for 10 min.
  c. Dilute 10 times with milliQ water.
37. Prepare PCR template (single cell).
  a. Heat 0.5% agarose in milliQ water (w/v) to completely dissolve.
  b. Add 1 mL of 0.5% agarose to 100 mm dish and spread.
  c. Cool to approximately 20°C–25°C.
  d. Incubate at 55°C until completely dry to make agarose-coated dish.
  e. Add 10–15 mL PBS to the agarose-coated dish.
  f. Spread approximately < 100 cells.
  g. Collect single cell with 1 μL surrounding PBS into 10 μL proteinase K working solution under an inverted stereo microscope.
  h. Incubate at 55°C for > 6 h.
  i. Heat at 95°C for 10 min.
38. Run the PCR with following conditions.

**Table:** PCR reaction master mix

Reagent	Amount
DNA template	1 μL
KOD	0.4 μL
Primer 1: human mtDNA forward (10 μM)	0.6 μL
Primer 2: human mtDNA reverse (10 μM)	0.6 μL
10 × KOD buffer	2 μL
2 mM dNTPs	2 μL
25 mM MgSO_4_	0.8 μL
MilliQ water	12.6 μL
Total	20 μL

**Table:** PCR cycling conditions

Steps	Temperature	Time	Cycles
Initial Denaturation	94°C	2 min	1
Denaturation	98°C	10 sec	30–40 cycles
Annealing/Extension	68°C	3 min	
Final extension	68°C	7 min	1
Hold	16°C	Forever

39. Digest the PCR products with *Ssp*I.
  a. Add 2 μL PCR product to 10 μL *Ssp*I working solution.
  b. Incubate at 37°C for > 1 h.
40. Separate the samples by electrophoresis with a standard protocol using 1.0–1.5% agarose gel.
41. Stain with EtBr solution for 1 h at approximately 20°C–25°C.
42. Observe the resulting fragments using a gel imager.

***Note:*** This RFLP analysis generates 1,854 bp- and 1,289 bp-fragment from PCR products of HeLa mtDNA and 143B mtDNA, respectively. If you use the cells genetically labeled with EGFP or mCherry, nuclear origin can be determined by PCR method using the EGFP or mCherry primers with the same condition of for mtDNA. In this case, Annealing/Extension time may shorten to 1 min. For identification of nuclear origin, you can use any other genetic markers.

## Expected outcomes

**Figure 9:**
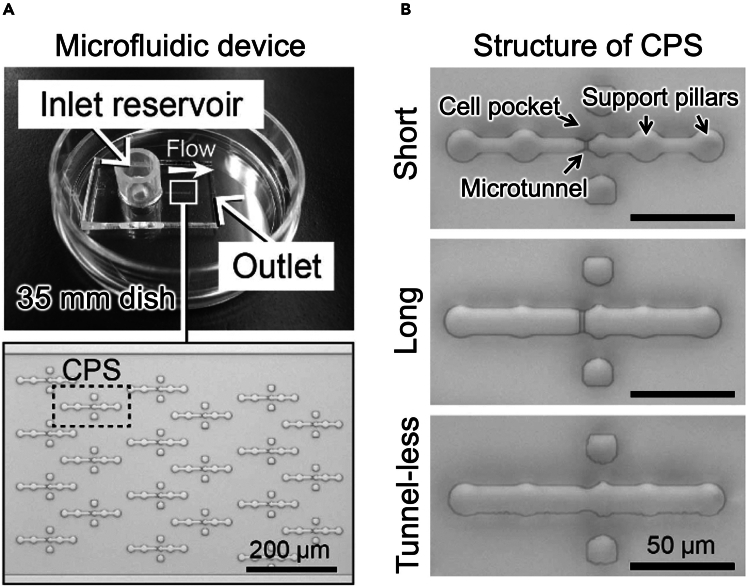
Summary of microfluidic device (A) Upper: whole image of microfluidic device. Lower: magnified view of main channel. In main channel, same type of CPSs (cell paring structures) are arrayed. Bar: 200 μm. (B) Images of CPSs with a short (4 μm-length) or long (10 μm-length) mirotunnel and without microtunnel. These different types of CPSs are made by combining certain first and second layers (see also Figure 2). Images are shown in the same magnification. Bars: 50 μm. The data has been previously published in Wada et al.,^1^ and reproduced here in agreement with publishers copyright policies. The article is licensed as CC BY.

**Figure 10:**
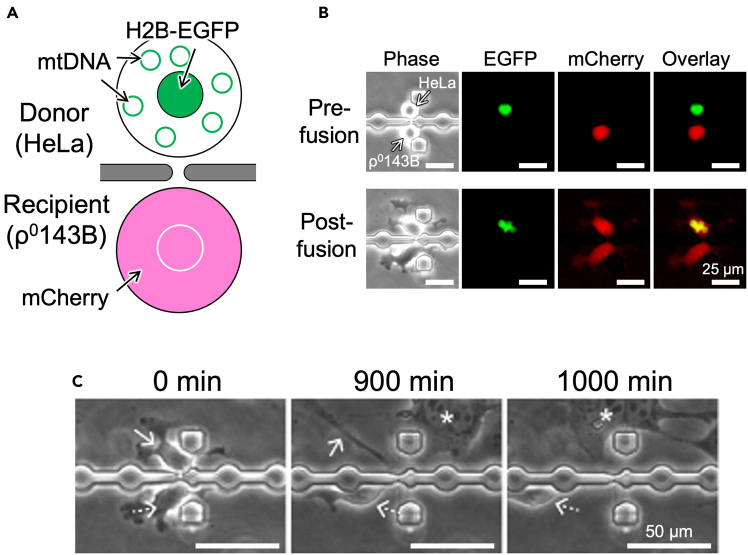
Achievement of partial cell fusion and following disconnection In this experiment, the microfluidic device having 105 CPSs with a short (4 μm-length) microtunnel was used. (A) Summary of the experiment. After trapping H2B-EGFP-expressing HeLa (donor) and mCherry-expressing ρ^0^143B (recipient) cells in CPSs, fusion medium was flowed for 1 h, then Pur selection medium was flowed for 1 day. In this scheme, mitochondria are omitted. (B) Fluorescent images of the fused cell pair. In this experiment, mtDNA was not visualized. Cytoplasmic mCherry was transferred from ρ^0^143B to HeLa cell, indicating achievement of partial cell fusion between them. H2B-EGFP and mCherry signals are presented in green and red, respectively. Phase: phase contrast image. Pre-fusion: immediately after cell trapping. Post-fusion: immediately after 1 h-flowing of fusion medium. Images are shown in the same magnification. Bars: 25 μm. (C) Time-lapse observation of the fused cell pair shown in B. Solid and dashed arrows represent the HeLa and ρ^0^143B, respectively. Asterisk indicates the cell moved from outside the CPS. The fused cells were spontaneously disconnected to recover into single cells during Pur selection medium flowing. Images are shown in the same magnification. Bars: 50 μm. Part of data has been previously published in Wada et al.,^1^ and reproduced here in agreement with publishers copyright policies. The article is licensed as CC BY.

Fine fabrication of the master mold is a key for the functionality of the microfluidic device. In particular, thickness of SU-8 (especially, second layer) and microtunnel position (i.e., precise alignment between first and second layers) are critical. Figure 9 shows summary of the microfluidic device. The microfluidic device can perform not only 1:1 cell fusion through a microtunnel but also subsequent disconnection (Figure 10), leading to non-invasive intercellular mitochondrial transfer.^1^ Notably, this unique cell manipulation does not require connecting to any external machines because of its self-standing property. These features represent practical advantages of the microfluidic device for generating transmitochondrial cybrids. More importantly, the microfluidic device provides the means for single mitochondrion transfer (i.e., mitochondrial cloning) and selection of tansmitochondrial cybrids under pyruvate/uridine-supplemented conditions.^1^^,^^6^ This suggests that the method using the microfluidic device has a potential to generate transmitochondrial cybrids with homoplasmic mtDNA mutations including respiratory dysfunctional ones.

**Figure 11:**
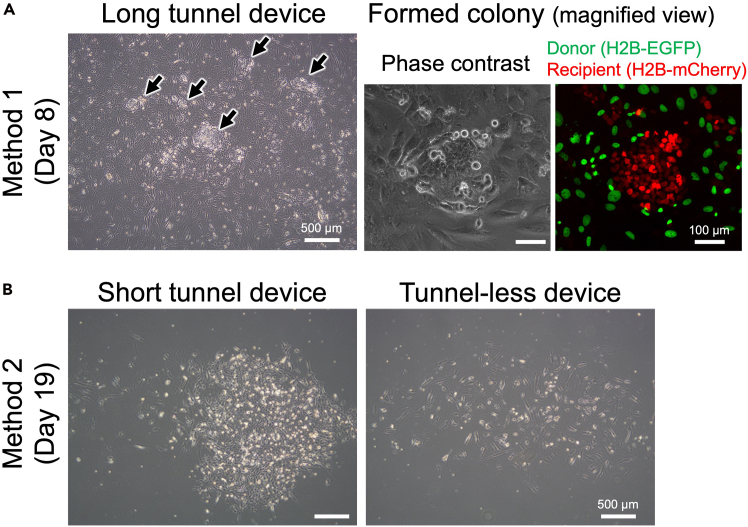
Colonies appeared during selective culture Experiments were carried out using H2B-EGFP-expressing HeLa (donor) and H2B-mCherry-expressing ρ^0^143B (recipient) cells. (A) A typical result of selection with Method 1 at day 8 (see also Figure 7). In this experiment, the microfluidic device having 1,260 CPSs with a long (10 μm-length) tunnel was used. Left: phase contrast image of the cells. Arrows indicates appeared colonies. At this point, donor cells were still alive. Subsequent selection with passages resulted in complete elimination of donor cells (data not shown). Bar: 500 μm. Right panels: magnified phase contrast and fluorescent images of a colony. Green: nuclei of donor cells (H2B-EGFP), Red: nuclei of recipient cells (H2B-mCherry). Images are shown in the same magnification. Bars: 100 μm. (B) Typical results of selection with Method 2 at day 19 (see also Figure 7). In these experiments, the microfluidic devices having 105 CPSs with a short (4 μm-length) microtunnel or without microtunnel (tunnel-less) were used. Images are shown in the same magnification. Bars: 500 μm. Left: phase contrast image of the cells in the experiment using short tunnel device. Right: phase contrast image of the cells in the experiment using tunnel-less device. While distinct colony appeared in short tunnel device, did not in tunnel-less device.

In this protocol, two methods for selection of transmitochondrial cybrids are shown (Method 1 and Method 2, Figure 7). Method 1 is an oxidative phosphorylation (OXPHOS)-independent method. Although this method requires to establish puromycin resistant gene-expressing cell as a mitochondrial donor, it is likely to generate transmitochondrial cybrids with respiratory dysfunctional mtDNA. Method 2 basically corresponds to a conventional OXPHOS-dependent method; however, it provides a simple and cost effective protocol. This method does not need enucleation process, and requires only a small amount of reagents. In both methods, the resulting cybrids are found as distinct colonies during selective culture (Figure 11). It is likely that both methods generate equivalent cybrids when mitochondria are transferred from the donors those which have same type of mtDNA; however, it is unclear whether the difference in these two methods influences the property of the resulting cybrids. Because, even among the cybrids generated with the same protocol, their properties are somewhat varied.

**Figure 12:**
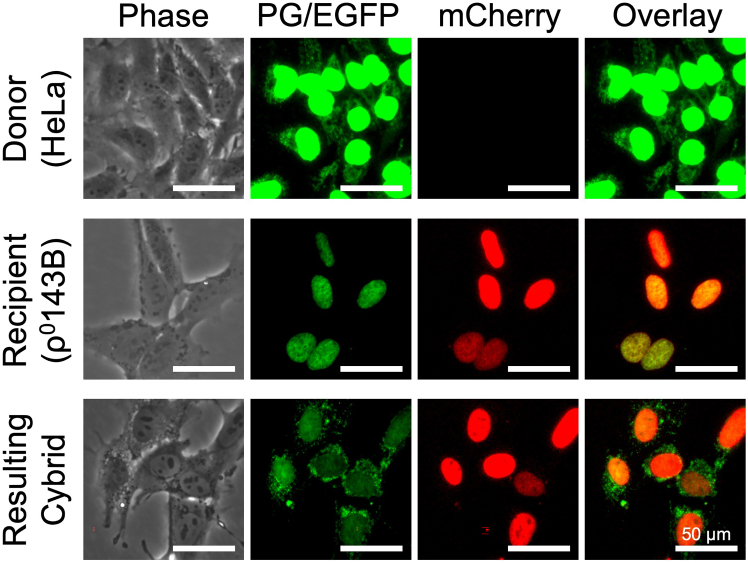
Confirmation of existence of mtDNA by fluorescent microscopic observation Fluorescent images of H2B-EGFP-expressing HeLa (donor), H2B-mCherry-expressing ρ^0^143B (recipient) and resulting cybrids. All cells were stained with PicoGreen. In this experiment, cybrids were generated using short (4 μm-length) tunnel device with Method 2 (see also Figure 7). Existence of cytoplasmic PicoGreen signals in the cybrids suggests repopulation of mtDNA. PG: PicoGreen. Images are shown in same magnification. Bars: 50 μm.

**Figure 13:**
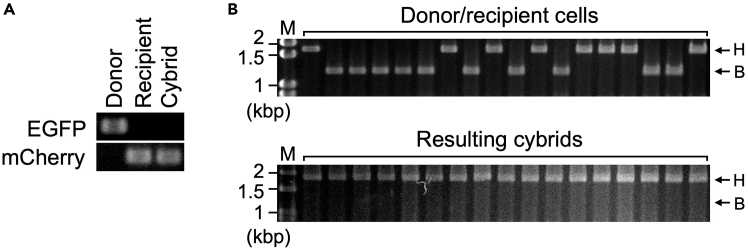
Confirmation of mtDNA repopulation by PCR-based methods In these experiments, the cybrids were generated from H2B-EGFP-expressing HeLa (donor) and H2B-mCheryy-expressing ρ^0^ 143B (recipient) cells using the microfluidic device having 1,260 CPSs with a long (10 μm-length) microtunnel. The cybrids were harvested with Method 1 (see also Figure 7). (A) Identification of nuclear origin by PCR. The resulting cybrids had mCherry gene but did not EGFP gene, indicating that the cybrids were derived from the recipient cell. (B) Identification of mtDNA origin by RFLP analysis in single cell samples. Upper panel: single cells from mixed suspension of donor and recipient cells. Lower panel: single cells from resulting cybrids. H: HeLa mtDNA (1,854 bp-fragment). B: 143B mtDNA (1,289 bp-fragment). M: size marker. Part of data has been previously published in Wada et al.,^1^ and reproduced here in agreement with publishers copyright policies. The article is licensed as CC BY.

This protocol also describes two simple methods for confirming mtDNA repopulation in the resulting cybrids. One is fluorescent observation-based method. In this method, the existence of mtDNA is detected as punctate cytoplasmic signals (Figure 12). The other is PCR-based RFLP analysis using *Ssp*I. This method complements the fluorescent observation-based method; the RFLP analysis can determine origin of the repopulated mtDNA in the cybrids. In this method, 1,854 and 1,289 bp-fragment is generated from the PCR products of HeLa and 143B mtDNA, respectively (Figure 13).

## Limitations

The microfluidic device cannot be applied to floating/non-adherent cells because the promotion of neurite-like process penetrating the microtunnel is essential to achieve the partial cell fusion.^8^ We have confirmed that cell fusion between ρ^0^143B cell and non-adherent cell such as K562 (lymphoblast) and THP-1 (monocyte) resulted in producing adhesive hybrids (data not shown). These hybrids may be used as a mitochondrial donor.

In this protocol, partial cell fusion is induced by HVJ-E-based method. Since this method cannot be replaced with other methods such as PEG-based method nor electrofusion, use of HVJ-E or other virus-derived components is essential. Unfortunately, the HVJ-E product (GenomeONE-CF) is discontinued. If you don’t have this reagent, it is needed to get an equivalent product (inactivated Sendai virus or extracted envelope components) from a laboratory doing research on this virus.

## Troubleshooting

### Problem 1

• Fail to generate ρ^0^ cells (related to Step 1).
• Complete elimination of mtDNA from the cells is critical for generation of ρ^0^ recipient cells, because incomplete mtDNA elimination results in endogenous mtDNA recovery.

### Potential solution

• EtBr concentrations: If the cells do not decrease mtDNA during the first week or result in incomplete mtDNA elimination after > 1 month in step 1a, the EtBr concentration is too low. When the cells abnormally expand or cell growth stops, the EtBr concentration is too high. Since proper EtBr conditions vary depending on cell types, it is strongly recommended to test wide-ranged concentrations (e.g., 20–1000 ng/mL).
• Cell cloning: When mtDNA recovers in step 1b, cell cloning before this step is an effective option to ensure generation of ρ^0^ cells.

### Problem 2

• Misalignment of the microtunnel and/or unclear exposure pattern of CPSs (related to Step 26).
• Fabricating the microtunnel at the precise position is important to efficiently promote partial cell fusion (cell fusion through a mirotunnel). Acceptable misalignment is approximately 0.5 μm. Fine CPS micropattern is also important for the functionality of the microfluidic device.

### Potential solution

• Prepare multiple master molds: Since the required accuracy in the micropatterns alignment for thick photoresist layer is beyond mask aligner performance, it is essential to select the fine master mold from the products. To reduce rework, it is recommended to make multiple SU-8-micropatterned Si wafers in each photomask setup. (e.g., more than ten Si wafers in both first and second layers).
• Putting an elastic sheet under the Si wafer: This increases contact between the photomask and the photoresist-coated Si wafer, leading to precise micropattern exposure in some cases.

### Problem 3

PDMS inflow into the inlet hole and/or insufficient bonding of the inlet reservoir (related to Step 11).

### Potential solution

• Even application of PDMS: One simple method is to transfer uncured PDMS from flat surface to the silicone tube. Specifically, after spread a small amount of uncured PDMS on a clean flat surface (e.g., bottom of plastic culture dish), gently put the silicone tube on it.
• Additional PDMS application: If a gap is formed in the joint of the inlet reservoir, apply a small amount of uncured PDMS from outside of the tube to fill the gap and bake it again to cure.
• Cut the silicone tube neatly: This ensures fabrication of the inlet reservoir without a gap. Use of a fine knife rather than a scissors is recommended.

### Problem 4

• Low cell allocation in CPSs (related to Step 22).
• Making enough number of 1:1 cell pairs in CPSs is important to ensure achievement of the partial cell fusion. If the additional centrifugation does not improve the cell allocation, it is recommended to refabricate the master mold with some modifications.

### Potential solution

• Increase the microchannel height: The microchannel height is a critical factor for smooth cell flow. If cell aggregations are often formed, increase the thickness of the second layer (i.e., spin coat SU-8 with a lower rpm).
• Reduce the microchannel height: If plural cells are trapped in one cell pocket, decrease the thickness of the second layer (i.e., spin coat SU-8 with a higher rpm).
• Change the CPS geometry: If no cells are trapped in the CPSs, the cells might pass through the cell pocket. In this case, reduce the gap behind the cell pocket; however, it also causes detour of the cells. Proper gap is required for efficient cell trap.

### Problem 5

• Low cell fusion efficiency (related to Step 26).
• Basically, the probability of the partial cell fusion is low; at most 4–5 cell pairs in 105-CPS device. However, if you never succeed in the partial cell fusion under a situation of enough cell allocation in CPSs, try following potential solutions.

### Potential solution

• Y-27632 and HVJ-E dosage: Higher concentration of Y-27632 and HVJ-E enhances cell fusion through a mirotunnel. We confirmed that at least up to 200 mM Y-27632 can be used for 143B cells.
• Expand the microtunnel width: Wider mirotunnel width increases cell fusion rate; however, it also increases the risk for nuclear migration passing through a microtunnel, resulting in failure of partial cell fusion. It is recommended at most 3 μm-width.

## Resource availability

### Lead contact

Further information and requests for resources and reagents should be directed to and will be fulfilled by the lead contact, Ken-Ichi Wada (wada.kenichi.833@m.kyushu-u.ac.jp).

### Technical contact

Technical questions on executing this protocol should be directed to and will be answered by the technical contact, Ken-Ichi Wada (wada.kenichi.833@m.kyushu-u.ac.jp).

### Materials availability

This study did not generate new unique reagents.

### Data and code availability

Original data have been deposited to Mendeley Data [https://doi.org/10.17632/zh7y93h78t.1].

## Acknowledgments

The authors thank the Laboratory for Animal Resources and Genetic Engineering, Center for Developmental Biology, RIKEN Kobe, for providing H2B-EGFP and H2B-mCherry vectors, RIKEN BRC for providing 143B/TK-neoR and HeLa cells, and Dr. Eiichi Soeda for providing pTK5 (this gene was introduced to 143B cell and used as 143B [TK+] cell in some experiments). This work was supported by the Japan Society for the Promotion of Science Grant-in-Aid for Scientific Research (JSPS KAKENHI), grant nos. JP26650068, JP16K07207, JP21H02467, and JP23K21304, and the Single Cell Project and Engineering Network Project from RIKEN.

## Author contributions

K.-I.W. conducted the experiments, analyzed the data, and wrote the manuscript. K.-I.W. K.H., Y.I., M.M., Y.H., and Y.Y. reviewed and edited the manuscript.

## Declaration of interests

The authors declare no competing interests.
