# Supplementary material for: Protocol for generation of transmitochondrial cybrids under pyruvate/uridine-supplemented conditions using a microfluidic device
Source: STAR Protoc. 2025 Jul 17;6(3):103953. doi: 10.1016/j.xpro.2025.103953 (PMC12296426; doi:10.1016/j.xpro.2025.103953)
Supplement: Document S1. Figures S1–S3 [file mmc1.pdf]

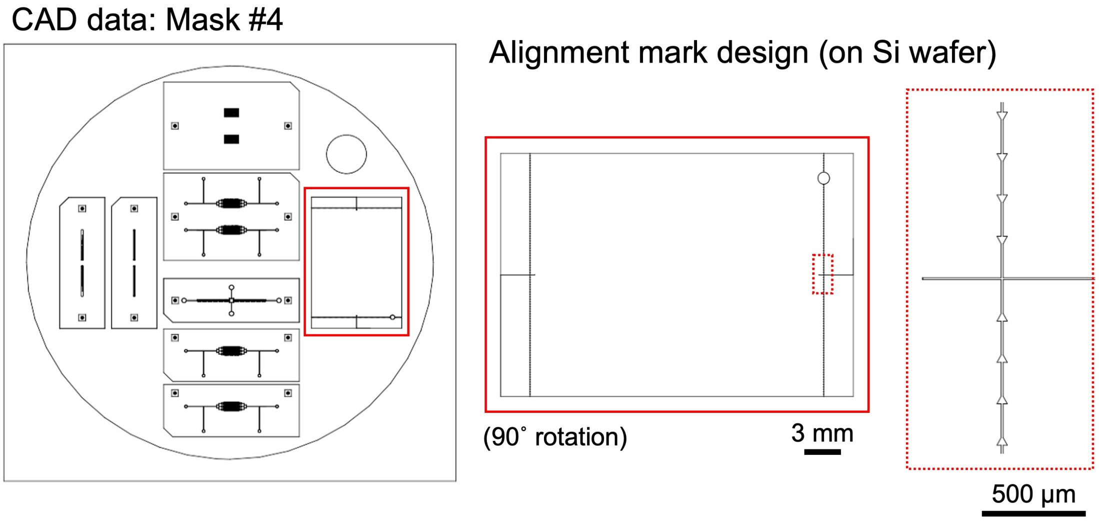

Figure S1: Summary of deposited CAD data of Mask #4, related to Step 3. Left: whole image of the CAD data. In this data, designs are drawn as 1 pix = 1 μm. Red box indicates the part of alignment mark design for Si wafer etching. Other designs are not used in this protocol. Right panels: magnified views of the alignment mark design.

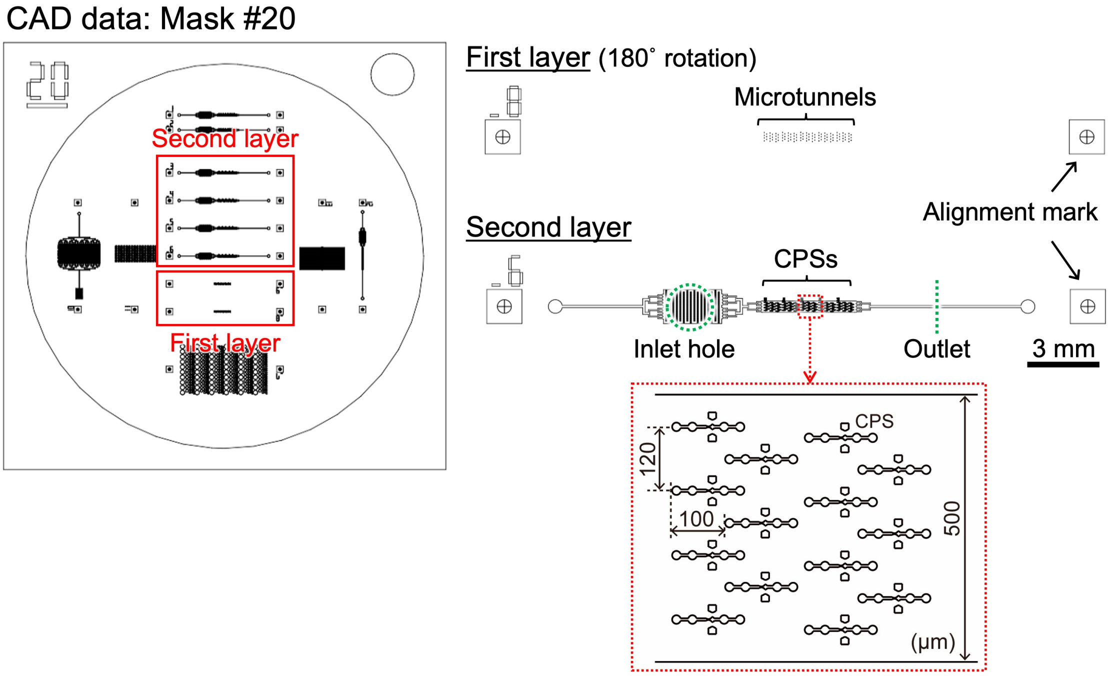

Figure S2: Summary of deposited CAD data of Mask #20, related to Step 3. Left: whole image of the CAD data. In this data, designs are drawn as 1 pix = 1 µm. Red boxes indicate the part of designs for first layer (microtunnels) and second layer (CPSs/main channel). Other designs are not used in this protocol. In this data, two types of microtunnel designs (0.5 and 1 µm-width microtunnels) and four types of CPS designs (4, 6, 10 and 16 µm-length microtunnels) are included. Combining these first/second layer designs, you can make master molds for microfluidic devices having 105 CPSs with a microtunnel of different width/length (see also Fig. 9). In this protocol, all master molds were made using 1 µm-width microtunnel design. Right panels: magnified views of first and second layers. Green dotted circle and line represent the region of inlet and outlet, respectively.

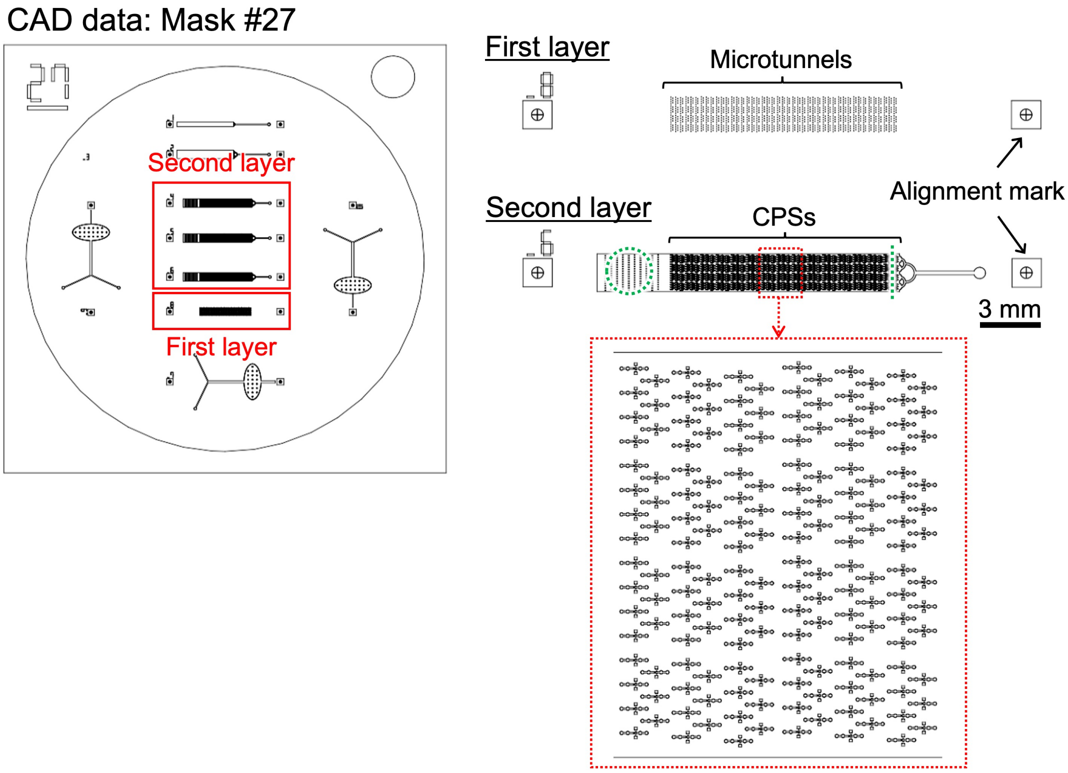

Figure S3: Summary of deposited CAD data of Mask #27, related to Step 3. Left: whole image of the CAD data. In this data, designs are drawn as 1 pix = 1 µm. Red boxes indicate the part of designs for first layer (microtunnels) and second layer (CPSs/main channel). Other designs are not used in this protocol. In this data, designs for 1 µm-width microtunnels and three types of CPSs (4, 6 and 10 µm-length microtunnels) are included. Combining these first/second layer designs, you can make master molds for preparation of microfluidic devices having 1,260 CPSs with a microtunnel of different length (see also Fig. 9). Right: magnified views of first and second layers. Green dotted circle and line represent the region of inlet and outlet, respectively.
